# Comparison of local infiltration analgesia and sciatic nerve block as an adjunct to femoral nerve block for pain control after total knee arthroplasty: A systematic review and meta-analysis

**DOI:** 10.1097/MD.0000000000006829

**Published:** 2017-05-12

**Authors:** Zhi Zhang, Qing Yang, Wenqi Xin, Yixuan Zhang

**Affiliations:** Department of Anesthesiology, Huaihe Hospital, Henan University, Kaifeng, China.

**Keywords:** local infiltration analgesia, meta-analysis, pain control, sciatic nerve block, total knee arthroplasty

## Abstract

**Background:**

To perform a meta-analysis to assess the efficiency and safety between local infiltration analgesia (LIA) and sciatic nerve block (SNB) when combined with femoral nerve block (FNB) for pain control following total knee arthroplasty (TKA).

**Methods:**

We systemically searched the following electronic databases for potentially relevant articles: Embase (1980–2017.01), Medline (1966–2017.01), PubMed (1966–2017.01), ScienceDirect (1985–2017.01), web of science (1950–2017.01) and the Cochrane Library. Only studies published in English that were accessible online were considered. Furthermore, we only considered studies that were published from 1966 to 2017. Only studies that met the following inclusion criteria were considered: (a) patients were adult human subjects who were set to undergo TKA; (b) the intervention was either SNB combined with FNB or LIA combined with FNB; (c) the outcomes of the studies, such as visual analog scale (VAS) scores, morphine consumption, length of stay and postoperative adverse effects, including the risk of nausea, vomiting and falls, were reported; (d) studies were either RCTs or non-RCT. Meta-analysis was performed using Stata 11.0 software. Modified Jadad score (7-points scale) which was based on Cochrane Handbook for Systematic Reviews of Interventions is used for assessment of RCTs. The Methodological Index for Nonrandomized Studies (MINORS) scale was used to assess non-RCTs with scores ranging 0 to 24. The synthesis of the outcomes for all studies was calculated as the weighted average rate by using a fixed or random effect model which depends on statistical heterogeneity. Systematic review registration number is CRD42017110661.

**Results:**

Three randomized controlled trials (RCTs) and 2 nonrandomized controlled trials (Non-RCTs), including 240 patients met the inclusion criteria. The present meta-analysis indicated that there were significant differences between groups in terms of visual analog scale (VAS) score at 12 hours (SMD = −0.337, 95% CI: −0.593 to −0.081, *P* =.010), VAS score at 24 hours (SMD = −0.337, 95% CI: −0.612 to −0.061, *P* =.017), morphine equivalent consumption at 24 hours (SMD = −0.371, 95% CI: −0.627 to −0.114, *P* = .005) and incidence of nausea (RD = 0.215, 95% CI: 0.078 to 0.353, *P* = .002) and vomiting (RD = 0.143, 95% CI: 0.026 to 0.260, *P* = .017).

**Conclusion:**

FNB combined with SNB provided decreased VAS scores and less morphine consumption at 12 and 24 hours compared with FNB combined with LIA in total knee arthroplasty. In addition, it was associated with lower risks of nausea and vomiting. We assessed the quality of the evidence as low to very low; therefore, our confidence in the effect estimate is limited, and the true effect may be substantially different from our estimates. Further studies should focus on surgeries that are known to be associated with significant postoperative pain, particularly surgeries where improved pain control may deliver significant clinical benefits through reduced morbidity, or cost-effectiveness benefits through faster rehabilitation and discharge. The present meta-analysis has the following limitations: (1) only 5 studies were included in the meta-analysis. Although all of them are recently published studies, the sample sizes are relatively small; (2) Functional outcome is an important parameter; however, owing to the insufficiency of relevant data, we failed to perform a meta-analysis on functional outcome; (3) The doses of anesthetics and the concomitant pain management regimes varied between the studies, which may have influenced the results; (4) The duration of follow-up was relatively short, which might have led to an underestimating of complications; and (5) publication bias present in the meta-analysis may have influenced the results.

## 1 Introduction

Total knee arthroplasty (TKA) is considered an effective procedure for the treatment of degenerative arthritis. However, patients who undergo TKA often experience moderate to severe postoperative pain.^[1]^ Appropriate postoperative pain control, which is crucial for early ambulation and better functional outcomes, is usually achieved following postoperative rehabilitation.^[2–4]^ Furthermore, optimal pain management may decrease the length of stay and the risk of adverse events, such as deep vein thrombus (DVT) and pulmonary embolism (PE). Postoperative pain management has been an interesting topic for a few decades and remains controversial. Femoral nerve block (FNB) provides analgesia to the anterior portion of the knee with few side effects, and requires lower opioid consumption; however, residual posterior pain may influence patient's satisfaction.^[5,6]^

Local infiltration analgesia (LIA) has been promoted for a few decades and shows excellent outcomes for pain relief after TKA.^[7,8]^ A mixture that comprises a long-acting local anesthetic, a nonsteroid anti-inflammatory drug and epinephrine is most commonly used in local infiltration. LIA is a promising method with fewer side effects that offers early mobilization without weakness of the quadriceps muscle.^[9,10]^ Therefore, it has been considered as a possible adjunct to FNB after TKA. However, fundamental research has shown that the knee joint is also innervated by the sciatic nerve; thus, FNB combined with sciatic nerves block (SNB) has become a growing practice in pain management following TKA, as it provides improved pain relief.

Currently, the optimal adjunct to FNB following TKA remains controversial. Meta-analysis was performed as the major statistical method in the present study. It strengthens statistical power and enlarges sample size by pooling results from published articles that could offer stronger evidence. Therefore, we performed a meta-analysis from random controlled clinical trials (RCTs) and non-RCTs to assess the efficiency and safety of LIA and SNB when combined with FNB for a patient undergoing TKA. The primary outcomes included visual analog scale (VAS) scores, morphine consumption, length of stay, and postoperative adverse effects, including the risk of nausea, vomiting, and falls.

## 2 Methods

This systematic review was reported according to the preferred reporting items for systematic reviews and meta-analyses (PRISMA) guidelines. Systematic review registration number is CRD42017110661. The study was approved by the ethics committee of Huaihe Hospital.

### 2.1 Search strategy

We systemically searched the following electronic databases for potentially relevant articles: Embase (1980–2017.01), Medline (1966–2017.01), PubMed (1966–2017.01), ScienceDirect (1985–2017.01), web of science (1950–2017.01), and the Cochrane Library. Only studies published in English that were accessible online were considered. Furthermore, we only considered studies that were published from 1966 to 2017. Gray academic studies were also identified from bibliographies of included studies. The following terms were used as Keywords

**Figure 1:**
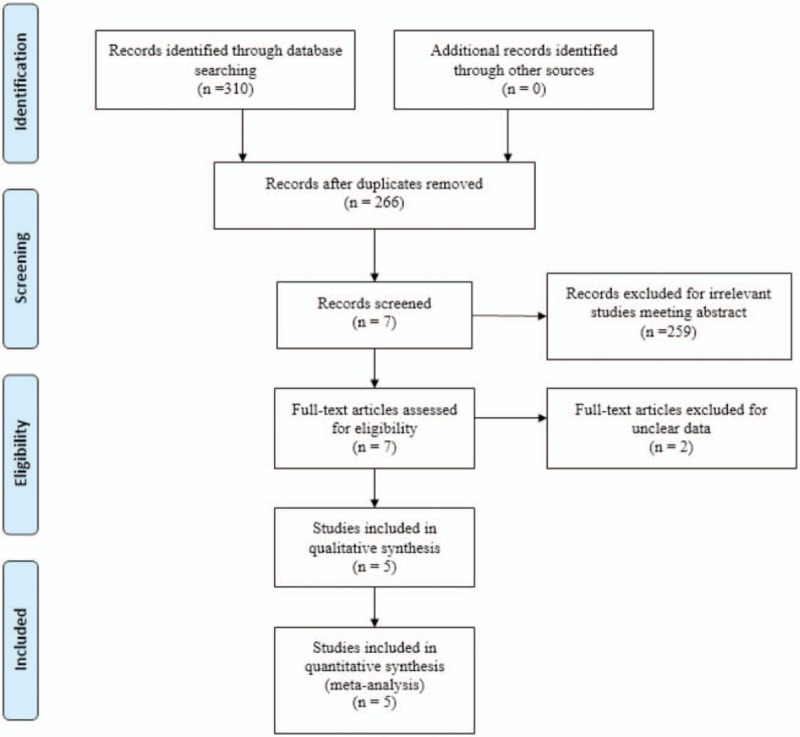
Search results and the selection procedure.

“Total knee replacement OR arthroplasty,” “local infiltration analgesia,” “sciatic nerve block,” and “pain control.” The keywords were used in combination with the Boolean operators AND or OR. The retrieval process is presented in Fig. 1.

### 2.2 Inclusion criteria and study selection

Only studies that met the following inclusion criteria were considered: (a) patients were adult human subjects who were set to undergo TKA; (b) the intervention was either SNB combined with FNB or LIA combined with FNB; (c) the outcomes of the studies, such as visual analog scale (VAS) scores, morphine consumption, length of stay and postoperative adverse effects, including the risk of nausea, vomiting and falls, were reported; (d) studies were either RCTs or non-RCT. Studies were excluded from the present meta-analysis if they contained incomplete data or were case reports, conference abstract, or review articles. Two reviewers independently reviewed the abstracts of studies identified for inclusion. After an initial decision, the full texts of the studies that potentially met the inclusion criteria were reviewed, and a final decision was made. A senior reviewer was consulted in cases where there was a disagreement.

### 2.3 Date extraction

A specific extraction was conducted to collect the following data from the included trials: patients’ general characteristics, the sample sizes of the control groups and intervention groups, the doses and types of anaesthetic used, concomitant pain, and duration of follow-up. Outcomes such as the VAS score at 12 and 24 hours, the morphine consumption at 12 and 24 hours, length of stay, and postoperative adverse effects (nausea, vomiting, and falls) were abstracted and recorded in a sheet. Data in other forms (i.e., median, interquartile range, and mean ± 95% confidence interval [CI]) were converted to the mean ± standard deviation (SD) according to the Cochrane Handbook. If the data were not reported numerically, we extracted these data using “Get Data Graph Digitizer” software from the published figures.

### 2.4 Quality assessment

Quality assessment of included studies was performed by 2 reviewers independently. The modified Jadad score (7-points scale), which is based on the Cochrane Handbook for Systematic Reviews of Interventions, was used for the assessment of RCTs. Studies whose scores were greater than 4 points were considered high quality. We prepared a “risk of bias” table including the following key points: random sequence generation, allocation concealment, blinding, incomplete outcome data, free of selective reporting, and other bias. Each item was recorded by a “Yes,” “No,” or “Unclear.” The methodological index for nonrandomized studies (MINORS) scale was used to assess non-RCTs with scores ranging from 0 to 24. Publication bias is the omission of unpublished trials from a meta-analysis. Trials are not published for a variety of reasons. Therefore, publication bias is an inherent weakness that exists in all meta-analysis. Selective reporting bias was tested using funnel plots.

### 2.5 Data analysis and statistical methods

All calculations were carried out with Stata 11.0 (The Cochrane Collaboration, Oxford, United Kingdom). Statistical heterogeneity was assessed based on the value of *P* and *I*^2^ using the standard chi-square test. When *I*^2^ > 50%, *P* < 0.1 was considered to be significant heterogeneous. The random-effect model was performed for meta-analysis; otherwise, the fixed-effect model was used. When possible, subgroup analyses were conducted to explore the origins of the heterogeneity. The results of dichotomous outcomes (postoperative adverse effects, including the risk of nausea, vomiting and falls.) were expressed as risk difference (RD) with a 95% confidence intervals (CIs). For continuous various outcomes (visual analog scale [VAS] scores, morphine consumption, length of stay), mean difference (MD), and standard mean difference (SMD) with a 95% confidence intervals (CIs) was applied for assessment. Subgroup analysis was conducted according to the anesthesia methods (general or spinal anesthesia).

## 3 Results

### 3.1 Search result

In the primary search, 310 articles were preliminarily reviewed. Finally, 5 studies met eligibility criteria of the present meta-analysis, 3 of them were RCTs^[11–13]^ and 2 were non-RCTs.^[14,15]^ Overall, the 5 studies included 119 patients in the SNB groups and 121 patients in the LIA groups

### 3.2 Study characteristics

Table 1 showed the basal line of participates in each study. All studies were published in English between 2014 and 2016 ranging in sample size from 34 to 65. There were 61 male patients and 273 female patients. Experimental groups received SNB combined with FNB and control groups received LIA combined with FNB. General anesthesia was applied in 3 trials and spinal anesthesia was used in 1 study. Four articles demonstrated that TKA was operated by the same senior teams. All participates in the included articles received postoperative concomitant pain management by opioids. All articles provided complete outcome data with a duration of follow-up ranging from 1 to 6 months.

**Table 1:**
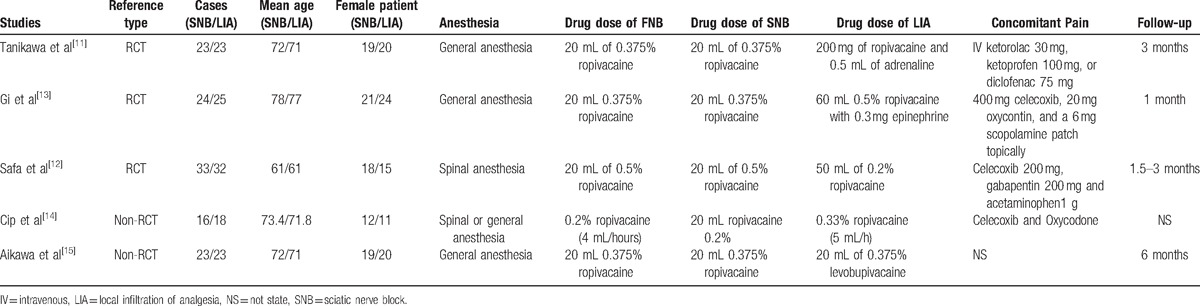
Trials characteristics.

### 3.3 Risk of bias assessment

A modified Jadad score based on the Cochrane Handbook for Systematic Reviews of Interventions was used to assess the RCTs (Table 2). All the RCTs^[11–13]^ provided clear inclusion and exclusion criteria, and suggested a methodology of randomization. Two^[12,13]^ of studies described that the randomization algorithm was generated by a computer program. Furthermore, 2 of the studies^[11,13]^ stated allocation concealment was achieved by the sealed envelope approach. Double blinding was provided in all RCTs. None of the studies attempted to blind the assessors. Each risk of bias item was presented as a percentage across all included studies. The percentage indicated the proportion of different levels of risk of bias for each item (Table 3). None of them performed intent-to-treatment analysis, thus a potential risk for type II statistical error would exist. The MINORS scale was used to assess non-RCTs by assigning scores ranging from 0 to 24 (Table 4).

**Table 2:**
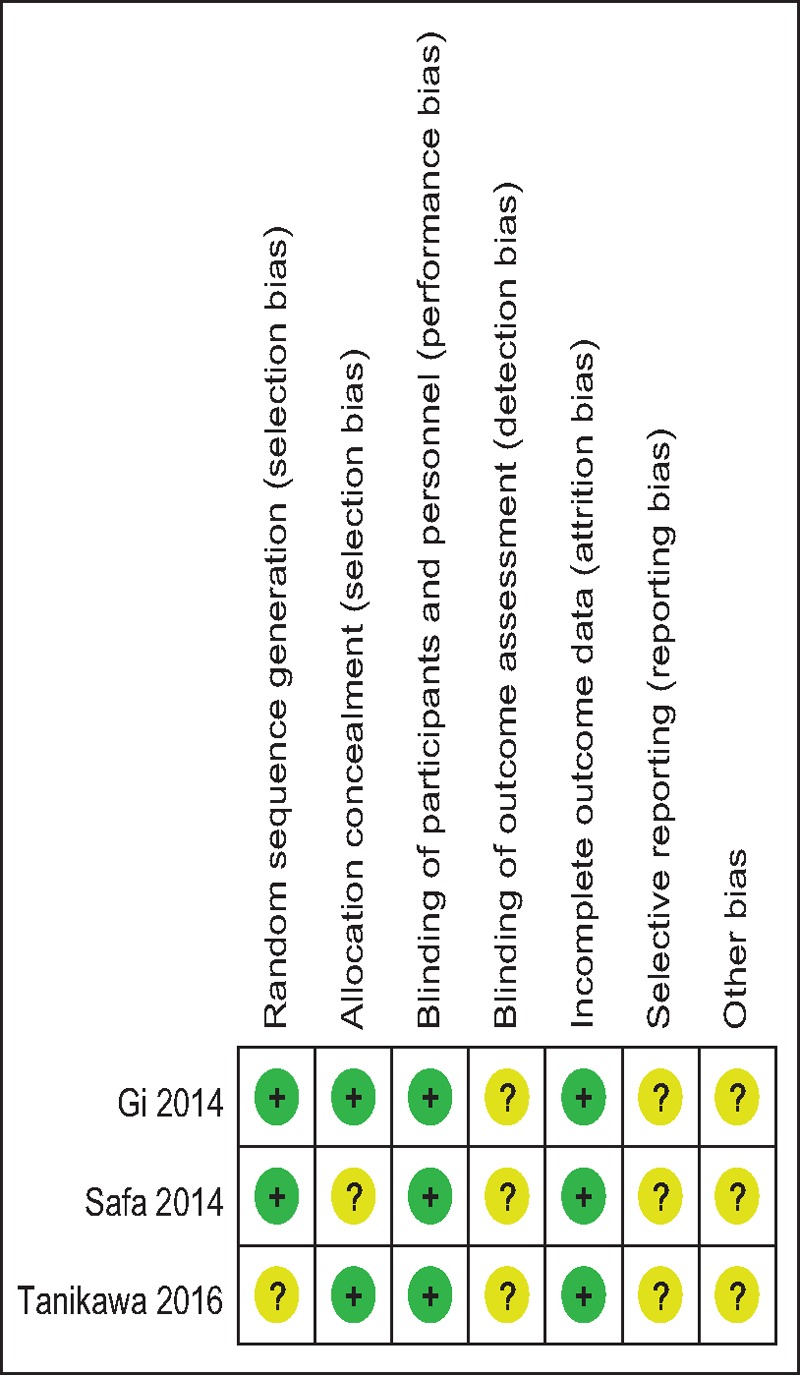
Methodological quality of the randomized controlled trials.

**Table 3:**
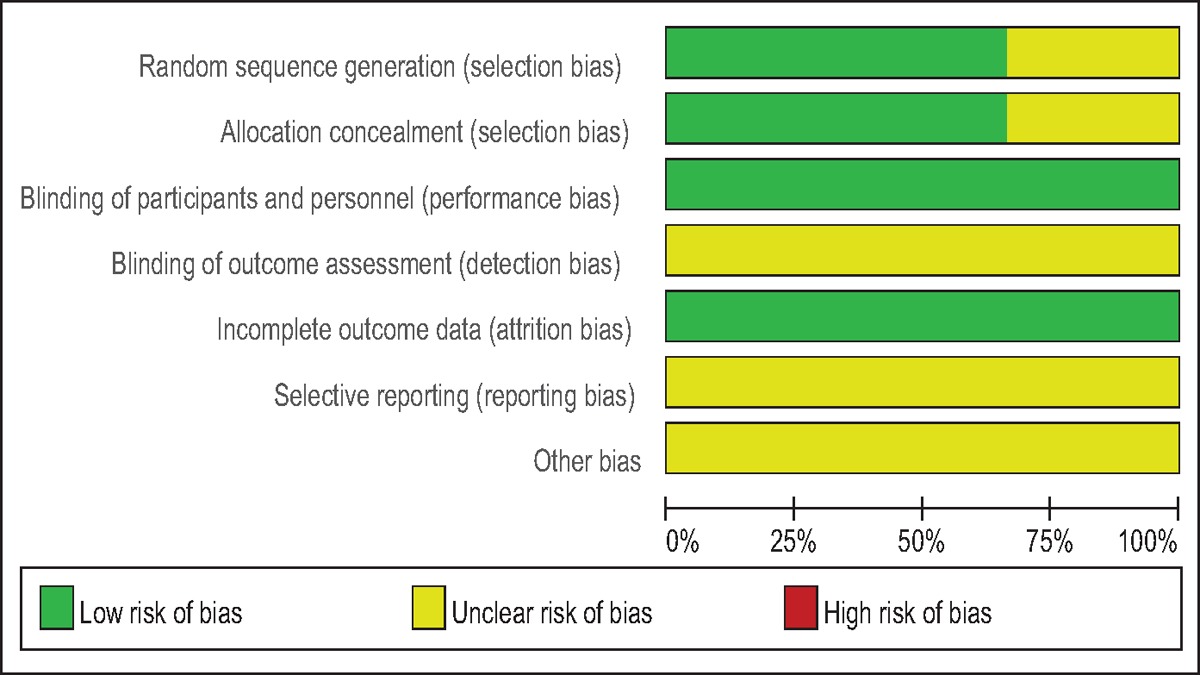
Risk of bias.

**Table 4:**
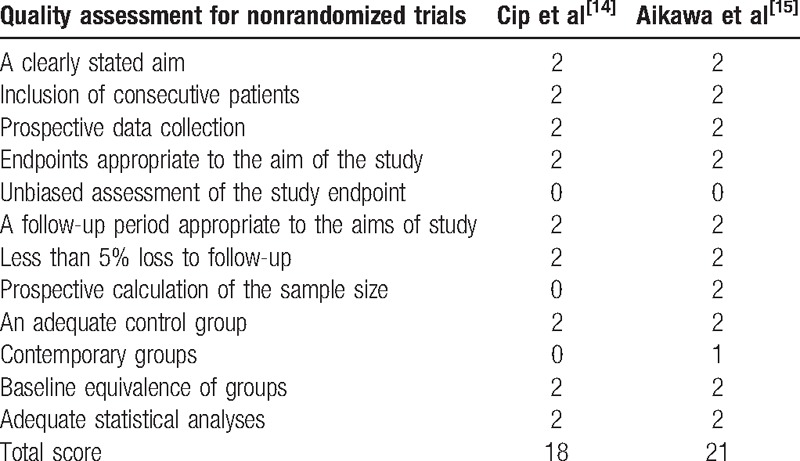
Methodological quality of the nonrandomized controlled trials.

### 3.4 Outcomes for meta-analysis

The analysis revealed that SNB combined with FNB could significantly decrease pain scores within 48 hours and reduce opioid consumption within 24 hours after TKA. Furthermore, there was a decreased risk of nausea and vomiting in the groups that received SNB combined with FNB.

#### 3.4.1 Pain scores at 12 hours

**Figure 2:**
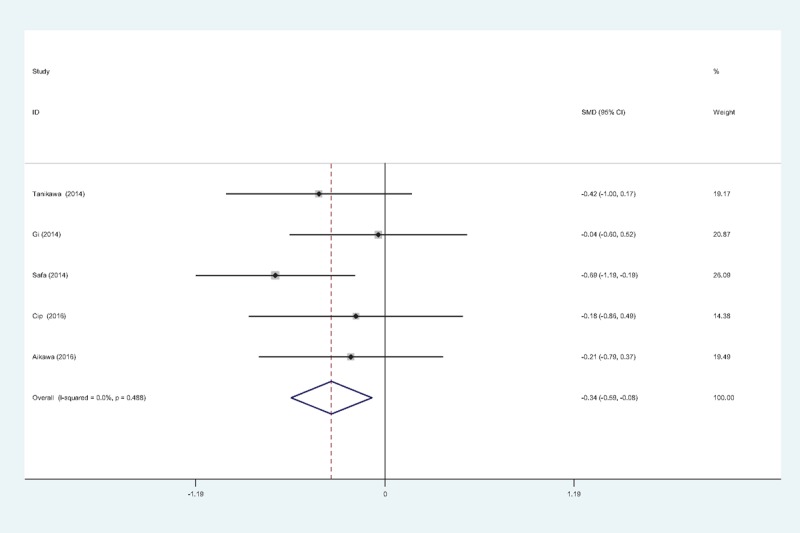
Forest plot diagram showing pain scores at 12 hours following TKA. TKA = total knee arthroplasty.

Five articles^[11–15]^ reported the outcomes of pain scores at 12 hours following TKA. There was no significant heterogeneity among the studies (χ^2^ = 3.43, df = 4, *I*^2^ = 0%, *P* = .448); therefore, a fixed-effects model was used. Pooled results demonstrated that pain scores at 12 hours in control groups were significant higher than in experimental groups (SMD = −0.337, 95% CI: −0.593 to −0.081, *P* = .010; Fig. 2).

#### 3.4.2 Pain scores at 24 hours

**Figure 3:**
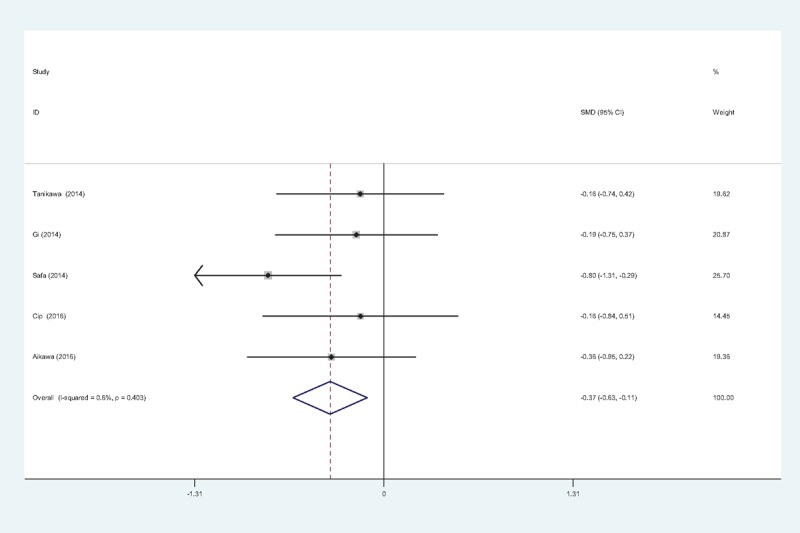
Forest plot diagram showing pain scores at 24 hours following TKA. TKA = total knee arthroplasty.

Five studies^[11–15]^ reported pain scores at 24 hours following TKA. There was no significant heterogeneity among the studies (χ^2^ = 4.03, df = 4, *I*^2^ = 0.6%, *P* = .403); therefore, a fixed-effects model was applied. Pooled results demonstrated that the pain scores at 24 hours in control groups were significantly higher than that in experimental groups (SMD = −0.371, 95% CI: −0.627 to −0.114, *P* = .005; Fig. 3).

#### 3.4.3 Pain scores at 48 hours

**Figure 4:**
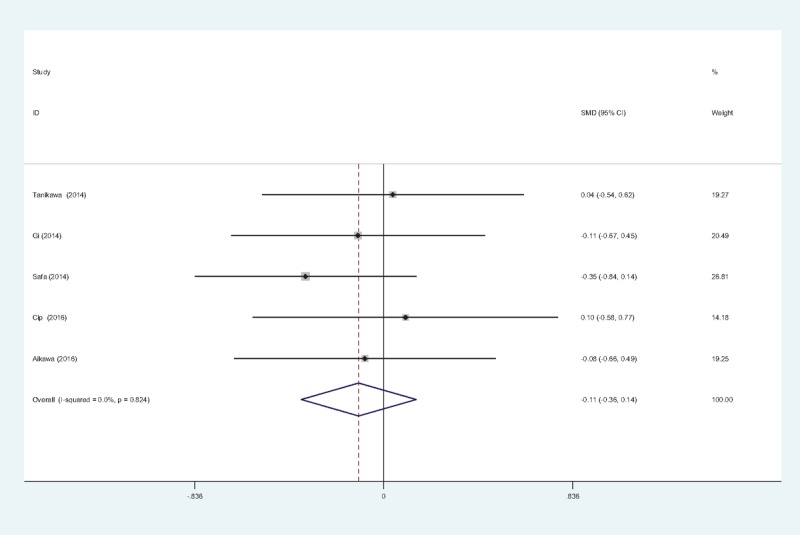
Forest plot diagram showing pain scores at 48 hours following TKA. TKA = total knee arthroplasty.

Five reports^[11–15]^ reported the outcomes of pain scores at 48 hours following TKA. There was no significant heterogeneity among these studies; therefore, a fixed-effects model was used (χ^2^ = 1.51, df = 4, *I*^2^ = 0%, *P* = .824). Pooled results demonstrated that pain scores at 48 hours in control groups was significantly higher than in experimental groups (SMD = −0.111, 95% CI: −0.365 to 0.143, *P* = .392; Fig. 4).

#### 3.4.4 Opioid consumption at 24 hours

**Figure 5:**
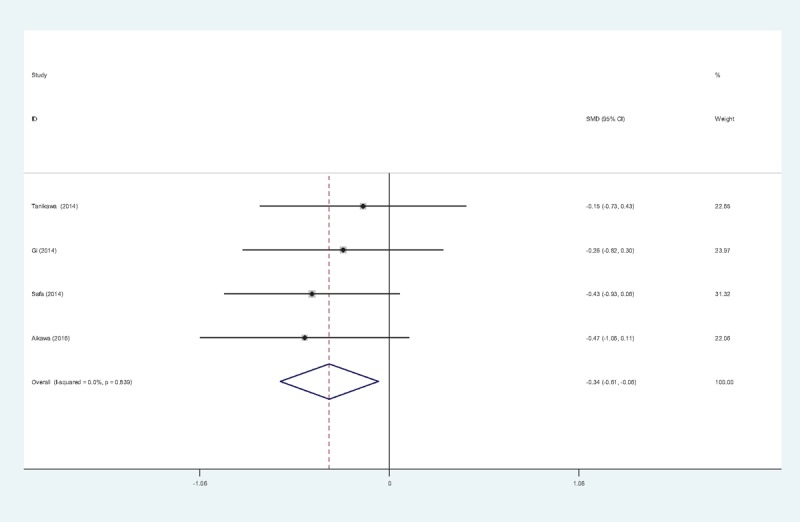
Forest plot diagram showing opioid consumption at 24 hours following TKA. TKA = total knee arthroplasty.

Opioid consumption at 24 hours after TKA was provided in 4 articles.^[11–13,15]^ No significant heterogeneity among these studies was found (χ^2^ = 0.84, df = 3, *I*^2^ = 0%, *P* = .839); therefore, a fixed-effects model was used. Opioid consumption at 24 hours in control groups was significantly higher than in experimental groups (SMD = −0.337, 95% CI: −0.612 to −0.061, *P* = .017; Fig. 5).

#### 3.4.5 Opioid consumption at 48 hours

**Figure 6:**
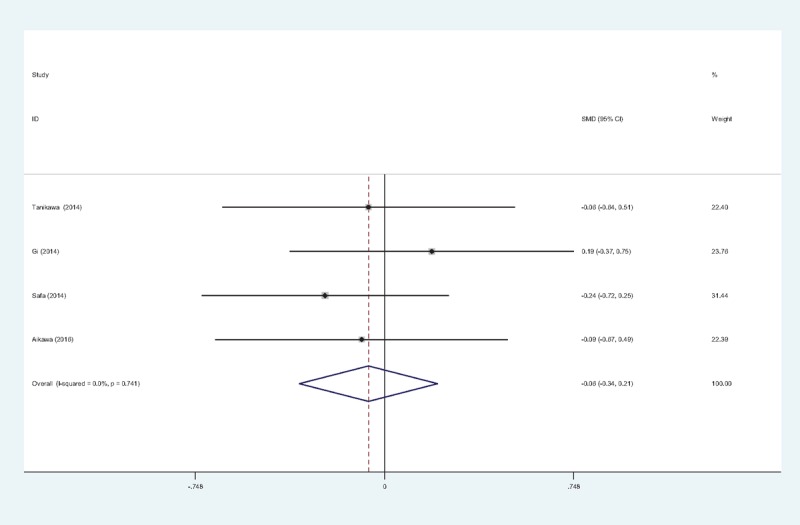
Forest plot diagram showing opioid consumption at 48 hours following TKA. TKA = total knee arthroplasty.

Four studies^[11–13,15]^ provided data regarding opioid consumption 48 hours after TKA. There was no significant heterogeneity among the pooled data (χ^2^ = 1.25, df = 3, *I*^2^ = 0%, *P* = .741); therefore a fixed-effects model was used. There was no significance between the 2 groups in opioid consumption 48 hours after TKA (SMD = −0.064, 95% CI: −0.338 to 0.209, *P* = .645; Fig. 6).

#### 3.4.6 Length of hospital stay (LOS)

**Figure 7:**
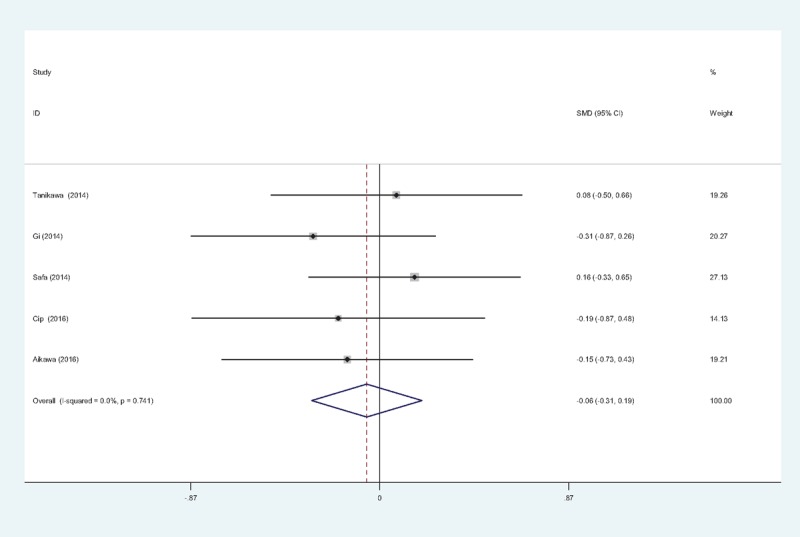
Forest plot diagram showing length of stay following TKA. TKA = total knee arthroplasty.

Five studies^[11–15]^ reported the length of hospital stay between groups. No significant heterogeneity was identified in the pooled results; therefore, a fixed-effects model was used (χ^2^ = 1.97, df = 4, *I*^2^ = 0%, *P = *.741). There was no significant difference between the 2 groups (SMD = −0.060, 95% CI: −0.313 to 0.194, *P = *.645; Fig. 7).

#### 3.4.7 The occurrence of nausea

**Figure 8:**
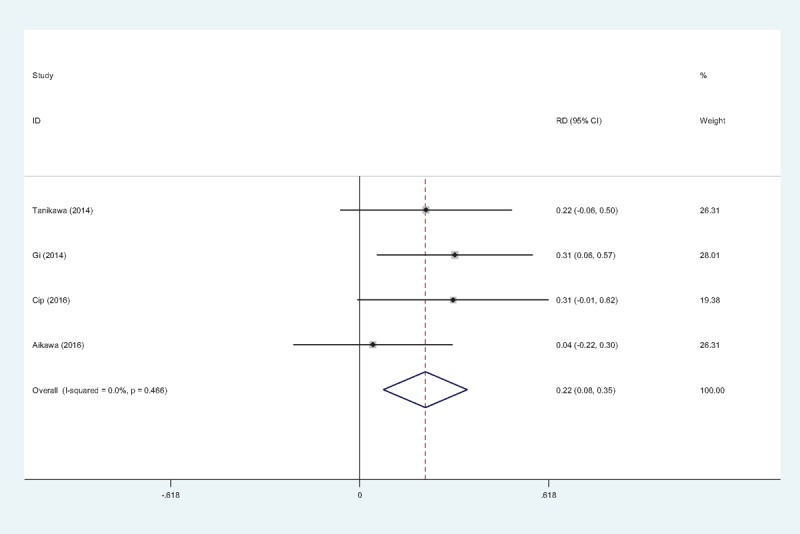
Forest plot diagram showing incidence of nausea following TKA. TKA = total knee arthroplasty.

The occurrence of nausea was provided in 4 studies.^[11,13–15]^ No significant heterogeneity among these studies was found; therefore, a fixed-effects model was used (χ^2^ = 2.55, df = 3, *I*^2^ = 0%, *P = *.466). There was a significant difference between the 2 groups (RD = 0.215, 95% CI: 0.078 to 0.353, *P = *.002; Fig. 8).

#### 3.4.8 The occurrence of vomiting

**Figure 9:**
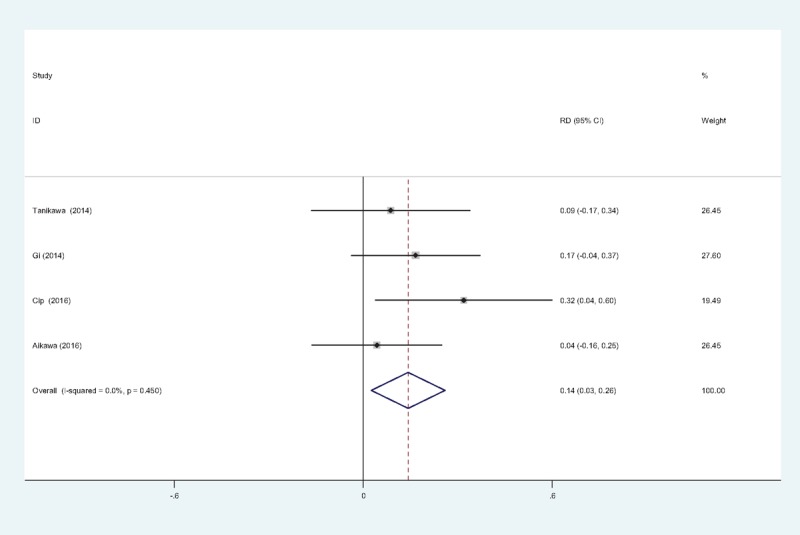
Forest plot diagram showing incidence of vomiting following TKA. TKA = total knee arthroplasty.

Four studies^[11,13–15]^ reported the incidence of vomiting. We found no statistical heterogeneity and a fixed-effects model was applied (χ^2^ = 2.64, df = 3, *I*^2^ = 0%, *P = *.450). The meta-analysis showed significant difference between groups. (RD = 0.143, 95% CI: 0.026 to 0.260, *P = *.017; Fig. 9).

#### 3.4.9 The occurrence of falls

**Figure 10:**
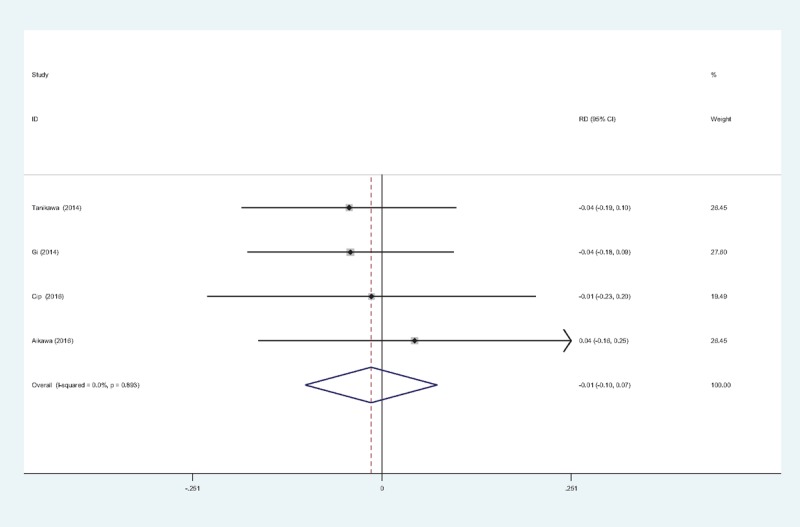
Forest plot diagram showing incidence of falls following TKA. TKA = total knee arthroplasty.

Four trials^[11,13–15]^ showed the incidence of falls. No statistical heterogeneity was found and a fixed-effects model was applied (χ^2^ = 0.62, df = 3, *I*^2^ = 0%, *P = *.893). The meta-analysis indicated that there was no significant difference between groups. (RD = −0.014, 95% CI: −0.102 to 0.073, *P = *.750; Fig. 10).

#### 3.4.10 Publication bias and subgroup analysis

**Figure 11:**
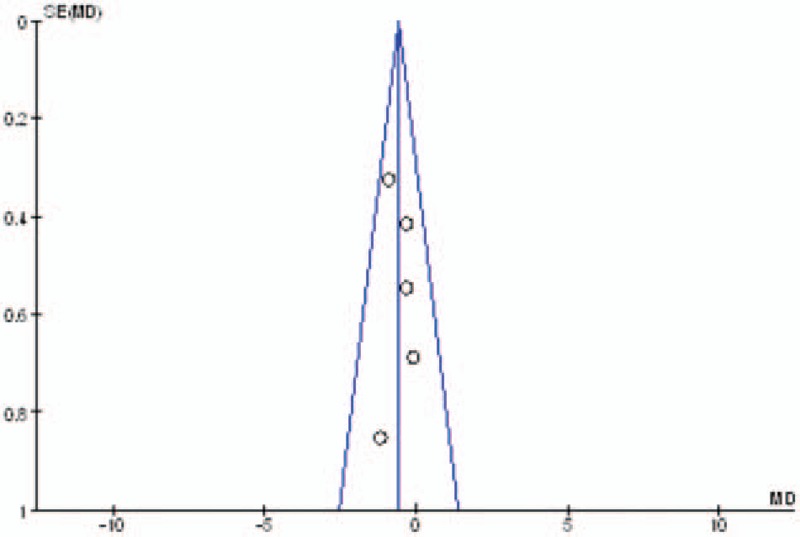
Funnel plot of pain score at 12 hours.

**Figure 12:**
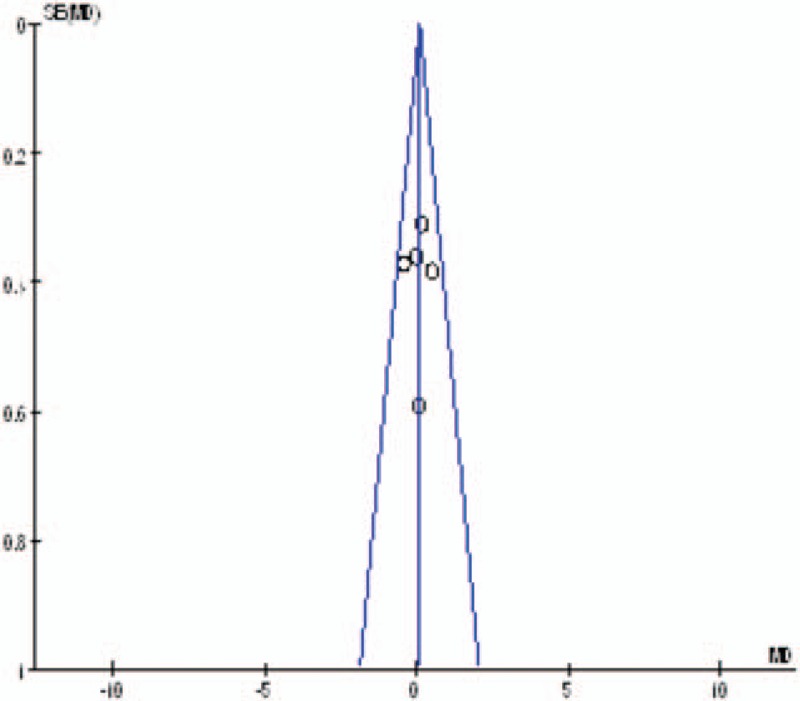
Funnel plot of pain score at 24 hours.

**Figure 13:**
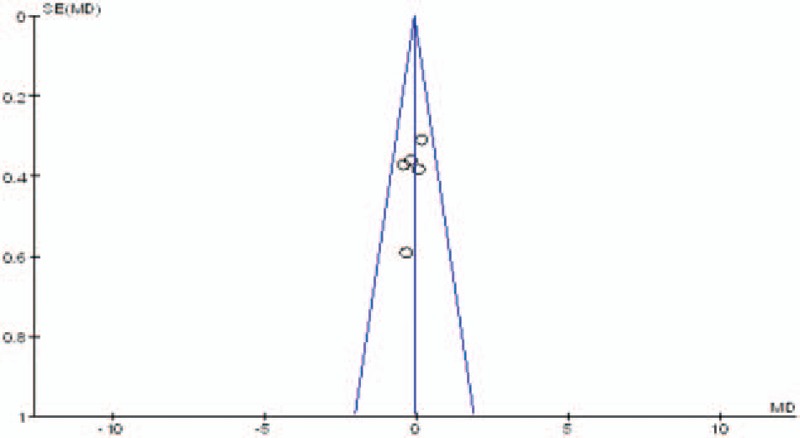
Funnel plot of pain score at 48 hours.

Publication bias was assessed by the most frequently reported outcome: the VAS scores. As shown in Figs. 11–13, the funnel plots were symmetrical, indicating a low risk of publication bias; however, publication bias could not be excluded, as the reliability of this kind of assessment was weak, especially as a low number of studies were included. The result of the subgroup analysis is presented in Table 5.

**Table 5:**
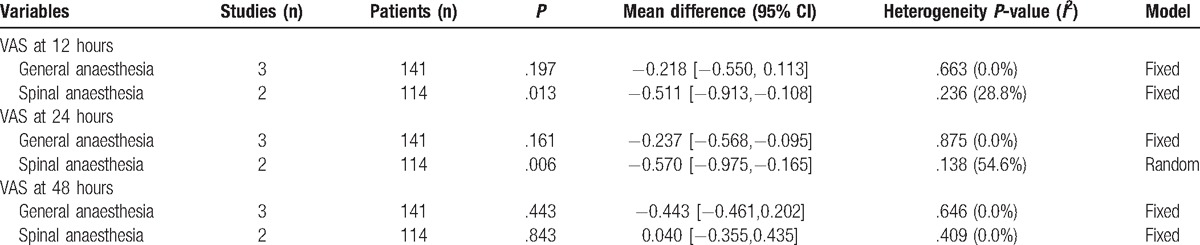
Subgroup analysis of the pain score at 12, 24, and 48 hours.

## 4 Discussion

To the best of our knowledge, this is the first systematic review and meta-analysis of published clinical trials to compare the effectiveness and safety of SNB combined with FNB versus LIA combined with FNB for pain control in total knee arthroplasty. The most important finding of the present meta-analysis was that SNB combined with FNB could significantly decrease pain scores within 48 hours and reduce morphine consumption within 24 hours after TKA. Furthermore, there was a decreased risk of nausea and vomiting in the groups that received SNB combined with FNB. All outcomes in this meta-analysis were evaluated using the GRADE system. The evidence quality for each outcome was low to very low (Table 6), which means that any effect estimate is uncertain. This finding may lower confidence in any recommendations.

**Table 6:**
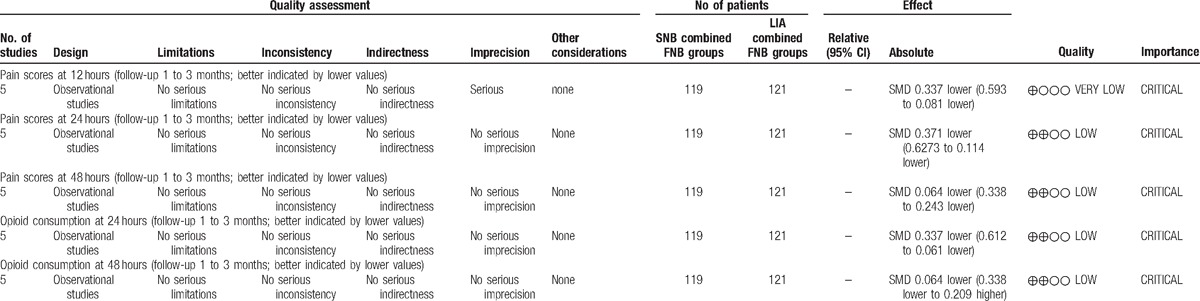
The GRADE evidence quality for main outcome.

Sciatic nerve block is a commonly used adjunct to femoral nerve block in TKA. Previous studies have shown its effectiveness in postoperative pain management compared with single FNB. Cook et al^[16]^ reported that the combined femoral and sciatic nerve block provided superior pain management in the early postoperative period after TKA. Pham Dhang et al^[17]^ performed an RCT to assess the benefit of using sciatic nerve block to improve analgesia after TKA, and suggested that the combination of continuous femoral and sciatic nerve block improved analgesia while decreasing morphine consumption and occurrence of postoperative nausea and vomiting. However, additional SNB may be associated with considerable side effects such as weakness in the quadriceps muscles, which results in an increased risk of postoperative falls.^[18]^ Furthermore, there is a risk of peripheral nerve injury, which has an incidence of 0.024% in patients who receive SNB.^[19]^ Sciatic nerve injury is also a common complication following TKA and its incidence is 1.3 to 2.2%.^[20,21]^ Therefore, LIA combined with FNB was suggested to achieve comparable pain control. Several types of local anesthetics have been administrated in TKA. Long-acting local anesthetics such as ropivacaine and levobupivacaine are commonly used. All the included studies used local ropivacaine whose concentration ranged from 0.2% to 0.5%. The present meta-analysis indicated that SNB combined with FNB had an analgesic effect that was superior to that of LIA combined with FNB at 24 and 48 hours following TKA. No significant difference regarding the incidence of falls were identified between groups.

Opioid consumption is considered as an objective method of measuring pain. Opioids-related adverse effects, such as nausea, vomiting, respiratory depression, and pruritus were reported in previous studies.^[22,23]^ Besides the side effects described above, drug dependence is also an important issue associated with opioid consumption that should be considered. Minimizing opioid consumption would improve patient satisfaction and expedite mobilization and rehabilitation. This study showed that there was decreased morphine consumption in the SNB combined with FNB groups compared with the LIA combined with FNB groups 24 hours after TKA; however, no significant difference was found between groups regarding morphine consumption 48 hours after TKA.

Nausea and vomiting are common side effects that are frequently associated with oral or intravenous morphine. Sufficient anaesthetic techniques can reduce morphine consumption and subsequently decrease the risk of complications. This study indicated that there was a decreased risk of nausea and vomiting in the SNB combined with FNB groups compared with controls. As only 5 studies were included in our meta-analysis, we did not perform investigations on dose dependence. Large sample sizes from high-quality RCTs are, therefore, needed.

Although, further evidence of the clinical benefits and cost effectiveness of SNB combined with FNB is required, the current data support the use of SNB combined with FNB to reduce postoperative pain in patients undergoing TKA. For clinicians, owing to the quality of evidence, the current data support the use of SNB combined with FNB for the management of postoperative pain. For policymakers, the current data do not permit firm estimates of the size of the effect owing to the low number of studies in the analysis and the low quality. For patients, SNB combined with FNB could significantly reduce pain, morphine consumption and adverse effects. Further evidence of clinical benefits, as well as cost-effectiveness, is required.

The present meta-analysis has the following limitations: (1) only 5 studies were included in the meta-analysis. Although all of them are recently published studies, the sample sizes are relatively small; (2) Functional outcome is an important parameter; however, owing to the insufficiency of relevant data, we failed to perform a meta-analysis on functional outcome; (3) The doses of anesthetics and the concomitant pain management regimes varied between the studies, which may have influenced the results; (4) The duration of follow-up was relatively short, which might have led to an underestimating of complications; and (5) publication bias present in the meta-analysis may have influenced the results.

### 4.1 Implications for practice and research

We assessed the quality of the evidence as low to very low; therefore, our confidence in the effect estimate is limited, and the true effect may be substantially different from our estimates. Further studies should focus on surgeries that are known to be associated with significant postoperative pain, particularly surgeries where improved pain control may deliver significant clinical benefits through reduced morbidity, or cost-effectiveness benefits through faster rehabilitation and discharge.

## 5 Conclusion

FNB combined with SNB provided decreased VAS scores and less morphine consumption at 12 and 24 hours compared with FNB combined with LIA in total knee arthroplasty. In addition, it was associated with lower risks of nausea and vomiting.
